# New York Letter

**Published:** 1882-07

**Authors:** 


### Domestic Correspondence.

ARTICLE IX.

NEW YORK, June 19, 1882.

*Editors* CHICAGO MEDICAL JOURNAL AND EXAMINER:

Our societies have held their last meetings for the season, and many physicians are beginning to start on their summer pilgrimage. A large number go to neighboring seaside or country resorts. A custom is springing up of sending a card to the profession in the city, notifying the brethren that such a one will be at such a place and hotel for the summer months. Dr. A., for example, will be at Ocean Hotel, Long Beach, during July and August. This is a practice which has long been adopted in a measure by German physicians. There can certainly be no objection to it. If a physician acts openly and straightforwardly, it is a poor code of ethics which he will transgress. One of the evils of the classic precepts of the old code was that it led to so many sneaking and evasive acts. This was strikingly shown in that satire, also classic, known as the "Experiences of a Successful Practitioner."

The past winter records the formation of two new societies. One of them, the Materia Medica Society, has been already referred to. The last one is known as the Practitioners' Society of New York. It is limited in number, but contains many prominent men. The president is Prof. William M. Polk. Other members are Dr. G. F. Shrady, Dr. R. F. Weir, Drs. Sexton, Hunter, Ward, and others.

At the last two meetings the subject of the removal of naso-pharyngeal tumors was discussed, Drs. Weir and Shrady each reporting a case. The points gained in the experience of these operators were valuable. One was that preliminary tracheotomy is the safest measure, even if the tumor is small. In Dr. Shrady's case the tumor was very large, and the hæmorrhage terrific. It was thought by Dr. Shrady that ligature of the external carotid would be a wise preliminary measure in future cases. Dr. Weir was inclined to think that in many cases hæmorrhage could be controlled by dividing the operation into two stages: First, that of exposing the tumor, then, after a few days, of removing it.

Dr. Hunter read a very practical paper upon vaginal and uterine discharges. In leucorrhœa from vaginitis he considered very hot water, as a rule, the best remedial agent. He deprecated the use of astringents as often keeping up the discharge. In leucorrhœa from cervicitis, injections were almost useless. Local applications must be made. Of these he preferred pure carbolic acid. The discussion drifted off to the subject of leucorrhœa in children. The fact was brought out that a urethritis in young boys and possibly leucorrhœa in girls might be due to a uric acid diathesis.

At the last meeting of the Academy of Medicine, Dr. F. D. Lente described a "New Form of Knee-joint Disease." It is quite doubtful whether it merits such a title. The disease follows slight injuries or wrenching of the knee. The patient suffers great pain at times, and walks with difficulty. There is tenderness, especially on the inner side of the knee, but no redness or swelling. It is not a hysterical joint. A number of cases were cited, and the speaker asserted that it was from this trouble that Prince Leopold recently suffered, thereby delaying his marriage. Dr. Lente did not venture to give the pathology. It seemed evident, however, that he described a trouble due to a sub-acute or chronic inflammation of some of the internal structures of the joint, probably the internal semi-lunar cartilage. The condition has been observed by others. The treatment is rest, with a splint. This brings about a cure in a short time.

Dr. Knight, surgeon in charge of the Hospital for Ruptured and Crippled, read a paper the same evening on "Static Electricity." He was very enthusiastic over the results obtained by it, especially in joint troubles. Dr. A. D. Rockwell thought it an excellent supplement to other forms of electricity. He referred to its tonic effects, and believed that when a patient was insolated and charged the electricity was diffused through the body. Dr. C. L. Dana had seen its tonic effects. He had had some speeially good results in local applications over the liver which had led him to inquire into its penetrating power. He believed that it was entirely superficial, and did not diffuse or penetrate except when sparks are drawn. Its effects, therefore, must be largely produced reflexly.

Dr. W. G. Morton said that he had found static electricity to be most powerful in all kinds of hysterical conditions, spinal irritation, etc.; next in rheumatic and painful joints, then in neuralgias, etc.

There has been considerable discussion here over Koch's alleged discovery of a bacillus tuberculosis. We want very much to have some sent over to us as has been done in England. I hear that an exhibition of them was given by some microscopical ( not pathological ) expert a short time ago. I have yet to meet anyone who puts faith in Koch's conclusions, though it seems impossible to deny the fact that he has found an organism. I have attempted at Satterthwaite's laboratory to demonstrate the bacillus. We had some beautiful specimens of tubercle. They were treated with methyl-blue and vesuvin, as described by Koch. No one at the laboratory, however, was ablc to distinguish them. We used a lower power (1-6) than is recommended by Koch. There are a number of obstacles which make it difficult to follow the Germans in their frequent epoch-making discoveries. It is generally found, for one thing, that they have been employing some new and unheard of dye which other people have to send long distances for, and then may not get it. By the time the rest of the profession is able to follow carefully the announced line of investigation, the original discoverer has achieved a reputation and perhaps got an official appointment. No better illustration can be found of the way in which a German pathologist can cover his tracks than in Erlich's recently announced method of showing bacilli tuberculosis. This remarkable man has a preciously new substance which he calls "anilin oil," in which, and in various other mixtures he baptizes the micrococci until they are inflated to nearly twice the size modestly given by Koch. The discovery of patriogenic bacteria is getting reduced to a fine art in Germany. It is a pity we cannot find some here.

It is a peculiar fact that the pursuits of neurology and jurisprudence should so inevitably develop personal differences. It took the Medico-Legal Society three months to elect its present officers, and now a war-cloud still hangs over it. The president, Mr. Bell, works for it with great energy, but he is said to be too autocratic for an ideal officer. At its last meeting the subject of Guiteau was introduced and discussed. The occasion was the petitition to President Arthur for a stay of proceedings, in order to allow Guiteau to be examined by a commission of experts. This petition has been extensively circulated, but not, I imagine, extensively signed. There was not a unanimity in the society as to whether Guiteau is insane. But it was voted that the society make no official announcement of its views.

The last two meetings of the Neurological Society have not been exhibitions of brotherly love. At the May meeting for election of officers, the two candidates previously nominated, withdrew in favor of Dr. Spitzka, who had not been previously nominated. Dr. Spitzka was elected after the second ballot. At the June meeting, a protest was presented signed by thirty-five members, against the way in which the president's election was secured. I do not understand exactly wherein the enormity lay, but the protest was put on the minutes after some warm interchange of views. Doubtless the matter will drop now, being sufficiently small to drop with no great noise. It has been suggested that it would be well to embody a certain well-known dramatic poem as part of the by-laws in all medical societies. This poem is known as "the Society Upon the Stanislow," and its spirit may be gathered from the following verse:

> "Now I hold it is not decent for a scientific gent,
> To say another is an ass—at least to all intent;
> Nor should the individual who happens to be meant,
> Reply by heaving rocks at him to any great extent."

The resignation of most of the clinical professors in the University Medical School took place some time ago. It is undoubtedly a loss to the school, and one not easily made good, since most of the gentlemen occupied the first position in the specialties they taught. There is, however, no particularly bad feeling about it. The rumor is that we are to have a Post-Graduate School. There were some attempts to organize a new medical school in connection with Cornell University. It is doubtful if this succeeds, as Cornell is not in the happiest state, financially or otherwise, just now. If it were possible to organize a post-graduate course which would secure and utilize the vast amount of clinical material here it would surely succeed. For a very large number of medical men come here every year to "brush up." This they do at present in a haphazard way, which is unsatisfactory and unprofitable.

We have had a "First Aid to the Injured Society" at work here for some time. It aims to teach men and women certain essentials in anatomy and physiology, and also what to do in case of injury, accidents, drowning, fracture, etc. The course consists of five lessons. It has been rather the mode to attend them the past winter, and a number of full classes have been diligently lectured to. The majority of the pupils are women, and a good many of them young ladies. There is a little book furnished for guidance. This book is rather better than the course. If the fever for elementary physiology and nursing should attack Chicago, I would advise you to meet it conservatively. The amount of knowledge which can be made practically useful in five lessons is not great. Few certainly can attain the quickness of thought and readiness of action which characterized *Punch's* medical student.

"What would you do, sir," asks *Punch*, "if you were called to see a man who had hung himself?"

"I would cut him down."

"Then what would you do?"

"I would cut him up."

I see by the German journals that similar societies, known as Samaritan Societies, have been organized in Berlin and other places. They have not met with unanimous favor there.

We are still having plenty of contagious diseases in New York. Typhus fever appeared here last February, and it does not disappear. Ten or fifteen cases are reported every week. Small-pox is a little more prevalent than typhus. We have some twenty new cases a week. I saw the report of the death by small-pox of a Boston actress not long ago. She had been opposed to vaccination. Such little memorials to Jenner's greatness are being furnished quite regularly. I saw a *mot* on the subject, which is too good to be kept in Paris—though it cannot be translated:

"Vérole et Variole," is the title; "Mlle. Duchand, de l' opera, etant morte de la petite vérole: 'C' est bien modeste,' dit Fontenelle."

